# The Future of the Army Medical Corps

**Published:** 1901-03-02

**Authors:** 


### The Hospital

A Journal of

The Medical Sciences and Hospital Administration.

Vol. XXIX.—No. 753] Edited by Sir Henry Burdett, K.C.B., and Solomon C. Smith, M.D., M.R.C.P. [March 2, 1901.

### THE FUTURE OF THE ARMY MEDICAL CORPS.

Distinct notice has now been given by Lord Raglan, in the House of Lords, and by Mr. Brodrick, in the House of Commons, that it is the intention of the Government to introduce "drastic" changes into the constitution of the Army Medical Corps, and at least to receive, whether or not they act upon, advice "from the heads of the medical profession and from all other possible sources," before the nature of the intended changes is finally determined. The word "drastic," according to Johnson, means "powerful, vigorous, efficacious"—three words which are not by any means synonymous—and "is used of a medicine that works with speed, as jalap, scammony, and the stronger purges." We know not whether the pharmacological associations of the word induced Mr. Brodrick to select it with the idea that it was in some way appropriate or complimentary to the medical profession, but it is perfectly easy to conceive of changes, emanating from the War Office, which might be powerful or vigorous without being in the least efficacious, or which, although operating as speedily as the stronger purges, might, like them, have no inconsiderable tendency to lower the stamina of the patient. However this may be, it is only too clear that the unfortunate Medical Department is about to be plunged again into the melting pot, and that it would be difficult for even the most accomplished prophet to predict the condition in which it will emerge. One thing only is certain, namely, that the "heads of the medical profession," whomsoever, in the estimation of the War Office, they may chance to be, are at least to be consulted. In the circumstances, whatever the "heads" of the medical profession may say and do, it seems incumbent upon the members of that profession to form, and to be prepared to impart and to justify, clear views as to the functions which the medical staff of the Army should be qualified and permitted to fulfil, and as to the position and authority essential to the effective discharge of the duties which ought to be imposed upon them.

In time of peace it may fairly be said that the duties of an army surgeon do not differ, in any material degree, from those of the medical attendant of any large body of civilians, such as a school or a great industrial establishment. The treatment of the actually sick, the inspection of fresh cases of illness as they are reported, the detection of malingering, the general regulation of the dietary, and the sanitary supervision of the common place of abode or of work include nearly all that has to be done, with the exception, of course, of the business of keeping necessary records. For all such purposes it is manifest that it is desirable to establish a close personal tie between the doctor and his actual or possible patients. The more he knows of them, the better qualified will he be to advise touching their welfare; and the more they know of him, if he be fit for his post, the greater will be their confidence in his skill and judgment. For all such purposes the positions of the regimental surgeon and assistant surgeon, as they were before the Crimean War, were ideal. Setting aside accidental instances of a square peg in a round hole, the doctor was everybody's friend, from the commanding officer to the drummer boy. In time of war all this is changed. Great general hospitals have to be established, a very large number of medical officers have to be prepared, in every action, to go up to the fighting line with their bearer companies to carry succour to the wounded, and an elaborate system of field dressing stations and field hospitals must be arranged for and fully provided with medical officers; and all this is quite beyond the powers of regimental officers. The old regimental system, useful as it was for the "domestic" doctoring of a regiment in time of peace, broke down for good and all in the Crimea as a means of dealing with the sick and wounded in war.

Many things have happened in this war which hardly could have occurred if generals in command had referred to, and had deferred to their medical officers more than they are in the habit of doing. Last year, in South Africa, a general kept the men under his command working hard at fatigue duty for two consecutive days, and on the night of the second day started them off to march for fifteen consecutive hours until they fell asleep as soon as they stood still, and were actually taken prisoners by the Boers in this somnolent condition. If men of this sort are to be put into commands they ought to have medical superintendents over them. Again, there can be no question that the outbreak of enteric fever in South Africa might have been at least diminished by precautions which a competent medical staff would have been able to suggest, and which a general of division possessed of commonsense would have been able to carry out in practice.

In the absence of these precautions its probable magnitude might at least have been foreseen and some adequate provision made for dealing with it. With reference to such general questions as these it is manifestly necessary to have a medical staff unconnected with regimental duty in a position to advise the commanding officer with regard to all general questions by which the health of the troops may be seriously affected, and to assist him in guarding against contingencies which his purely military knowledge would scarcely assist him to foresee. Such an organisation, combining staff and regimental officers and capable of being reinforced in any great war by a reserve force of *quasi*-civilian surgeons holding definite relations with the Army, would probably afford the best solution of questions which events have rendered urgent.

